# Virtual RAMPPS - an Online Simulated Teaching Method of the RAMPPS Model

**DOI:** 10.1192/bjo.2026.11810

**Published:** 2026-06-30

**Authors:** Chinwe Okeke, Ramy Teama, Louise Connor, Daniel Whitney

**Affiliations:** TEWV NHS Foundation trust, Middlesbrough, United Kingdom

## Abstract

**Aims:**

Virtual RAMPPS - An Online Simulated Teaching Method of the RAMPPS Model

**Methods:**

We delivered two separate half day courses, each including three scenarios to groups of Foundation Trainees, GP Trainees, Core Trainees and Trust Grade Doctors.Sessions were delivered on MS Teams and participants could answer next steps in their assessment/management anonymously using Slido.

Physical health emergencies covered included: Ligature Strangulation

Clozapine Induced Bowel Obstruction

Wernicke’s Encephalopathy

Neuroleptic Malignant Syndrome

Opioid Overdose

Venous Thrombo-Embolism

There was opportunity for debrief and discussion at the end of each clinical scenario, where personal and systemic factors affecting management of the cases were addressed along with questions from participants.

**Results:**

The study results were derived from qualitative feedback obtained from participants, which was analyzed to identify distinct themes regarding the simulation training. The findings were categorized into two primary domains: **Personal Factors** and **Systemic Factors**.

Under Personal Factors, the analysis highlighted the importance of early recognition, familiarity with guidelines, and case-specific confidence, which directly influenced the management of specific clinical scenarios such as cardiac problems, hypo- and hyperglycaemia, wound management, and overdoses.

Systemic Factors focused on operational elements, including resources, staffing, team knowledge, and the availability of senior psychiatric colleagues and nursing teams.

The results also mapped the complex logistical challenges faced by staff, such as the practicalities of transferring patients to acute hospitals, remote management, access to patient background information, and the specific limitations of medical provisions available on-site.

**Conclusion:**

Positive feedback demonstrates Virtual RAMPPS is a valuable learning tool, helps with personal factors like knowledge and skills, but also develops participants ability to deal with systemic factors. We intend to expand the course to include additional cases. Consideration will be made into expanding the audience to include physical healthcare practitioners and wider MDT.

